# A Case of Turner Syndrome with Concomitant Transient Hypogammaglobulinaemia of Infancy and Central Diabetes Insipidus

**DOI:** 10.4274/Jcrpe.880

**Published:** 2013-03-21

**Authors:** Hüseyin Anıl Korkmaz, Behzat Özkan, Filiz Hazan, Muammer Büyükinan, Tanju Çelik

**Affiliations:** 1 Dr. Behçet Uz Children Disease and Surgery Training and Research Hospital, Department of Pediatric Endocrinology, İzmir, Turkey; 2 Dr. Behçet Uz Children Disease and Surgery Training and Research Hospital, Department of Genetics, İzmir, Turkey; 3 Dr.Behcet Uz Children Disease and Surgery Training and Research Hospital, Department of Pediatrics, İzmir, Turkey

**Keywords:** Turner syndrome, hypogammaglobulinaemia, central diabetes insipidus, mental retardation

## Abstract

Turner syndrome (TS) is a genetic disorder that affects development in females and is characterized by the complete or partial absence of the second sex chromosome, or monosomy X. TS is associated with abnormalities in lymphatic and skeletal development, in growth, and in gonadal function. Cardiac and renal malformations and a number of specific cognitive findings may also be encountered in these patients. An increased risk for hypothyroidism, sensorineural hearing loss, hypertension, and other problems has also been reported. We present the case of a patient with TS accompanied by transient hypogammaglobulinaemia of infancy (THI) and central diabetes insipidus, which we believe is the first reported TS patient with these concomitant disorders.

**Conflict of interest:**None declared.

## INTRODUCTION

Turner syndrome (TS) results from complete or partial monosomy of the X chromosome and may occur in nonmosaic or mosaic forms, with or without the presence of a normal 46,XX or, occasionally, 46,XY cell line. Ninety-nine percent of fetuses with TS do not reach term. The 1% that survive demonstrate, to different extend, a wide spectrum of physical and neuropsychological characteristics of the syndrome, such as short stature, ovarian dysgenesis (causing sexual infantilism and infertility), lymphedema, cardiovascular defects, renal malformations, and hearing loss (1). Individuals with TS may also exhibit social and behavioral problems as well as cognitive deficits affecting nonverbal learning abilities and visuospatial skills (2). Recent neuropsychological investigations have revealed specific impairments in cognitive functioning in TS. The most commonly reported deficits relate to visual-spatial processing and visual memory and often manifest as ?difficulties in performing spatial and numerical tasks (2,3). Herein, we present the case of a female TS patient with transient hypogammaglobulinaemia of infancy (THI) and central diabetes insipidus.

## CASE REPORT

A 1-year-old female patient born at term to consanguineous parents was admitted to our hospital on two different occasions with a medical history of pneumonia and on three occasions for middle ear infections. Anthropometric measurements at the age of 1 year revealed a weight of 7.8 kg (<3rd percentile), a height of 70 cm (<3rd percentile), and a head circumference of 41 cm (<3rd percentile). No abnormalities were noted in the physical examination. Serum immunoglobulin levels were assessed on three occasions and the values were found to change with increasing age (Table 1). The antibodies resulting from vaccination [anti-diphtheria immunglobulin G (IgG), anti-tetanus IgG and anti-polio virus IgGs] were all detected. Values obtained for T cell counts [total lymphocytes 6.2 x 109/l, CD3:68% (55-82), CD4:56% (27-57), CD8:15% (14-34), CD19:26% (9-29), CD16+56:7% ?(10-22)] and phytohaemagglutinin-induced proliferation were normal. The nitroblue tetrazolium test was negative. Specific serologic tests for human immunodeficiency virus, cytomegalovirus, Epstein-Barr virus, rubella virus, toxoplasma gondii and parvovirus were negative. After performing multiple diagnostic studies to exclude other hematologic, immunologic and infectious disorders, the patient was diagnosed as a case of ?THI. Intravenous immunoglobulin (IVIG) in a dose of 1 g/kg/dose (500 mg/kg/every 3 weeks) was administered daily for a period of two months. When the patient was 2.5 years old, she developed polyuria and polydipsia. After exclusion of diabetes mellitus, hypokalemia and hypercalcemia, the patient underwent a water deprivation test to investigate the possibility of a diagnosis of diabetes insipidus. Water intake was restricted after starting the test in the morning. All blood samples for serum osmolality, BUN, creatinine, and sodium levels were drawn from an indwelling catheter. Urine samples for density and osmolality were collected every hour. At the beginning of the water deprivation test, the serum osmolality and sodium level were 288 mOsm/kg and 140 mmol/L, respectively, and the urine osmolality was 96 mOsm/kg. The water deprivation test was ended after eight hours because the patient lost 6% of her body weight and developed hypotension and tachycardia. At the end of the water deprivation test, the serum osmolality and sodium levels were 308 mOsm/kg and 150 mmol/L, respectively, and the urine osmolality was 122 mOsm/kg. Aqueous vasopressin (1U/m2) was given subcutaneously. Urine osmolality increased to 254 mOsm/kg during the hour following vasopressin administration. The patient was diagnosed to have central diabetes insipidus. Intranasal desmopressin was given by nasal tube. Magnetic resonance imaging of the brain revealed no abnormalities and posterior pituitary bright spot in magnetic resonance imaging of the pituitary gland was absent. Evaluation of the neuromotor development (the Bayley scales of infant development) was done at age 4 years and the patient’s psychomotor developmental index was found to be below normal. The patient was found to have difficulties especially in fine-motor coordination and language skills. Her audiological evaluation revealed normal findings. Karyotype analysis, which was performed due to the neuromotor developmental delay, revealed a 45,X0 pattern. Detailed physical examination demonstrated a low hairline, small lower jaw, webbed neck, cubitus valgus, and short fourth metacarpal.

## DISCUSSION

To our knowledge, this case is the first report of TS concomitant with THI and central diabetes insipidus. There are rare case reports of TS with THI in the literature. THI is originally defined as a physiological maturation defect of IgG production that occurs at 3-6 months of age and lasts until 18 to 36 months of age. The majority of children with THI may be asymptomatic, but children with recurrent infections have been incidentally detected to have THI (4). Our patient had a history of presenting to hospital on two occasions with pneumonia requiring hospitalization and on three occasions with middle ear infection. THI, which is a common cause of hypogammaglobulinemia in infancy, may also present with reduced serum Ig levels (4). In our patient, we found reduced levels of serum IgG, IgM, and IgA according to defined norms for age. Immunological disturbances associated with reduced levels of serum IgG and IgM, increased IgA, and decreased levels of circulating T- and B-lymphocytes have previously been described in TS patients. However, the results have not been conclusive (5,6,7,8,9). According to other authors (10), anatomic anomalies at the cranial base may result in alteration in the angle of the Eustachian tube, leading to the high incidence of recurrent acute otitis media. As immunological derangements and anatomic anomalies seem to be common in TS, an immunological deficiency could also be a potential cause of the ear problems and hearing impairment. Our patient had a diagnosis of THI and received IVIG treatment.

Central (hypothalamic, neurogenic, or vasopressin-sensitive) diabetes insipidus can be caused by a variety of disorders, such as: disorders of vasopressin gene structure; accidental or surgical trauma to vasopressin neurons; congenital anatomical hypothalamic or pituitary defects; neoplasms; infiltrative, autoimmune, and infectious diseases affecting vasopressin neurons or fiber tracts; and increased metabolism of vasopressin. In approximately 50% of children with central diabetes insipidus, the etiology is not apparent (11). Central diabetes insipidus has previously been described in a few cases of TS, but the results have not been conclusive (12,13). One child with septo-optic dysplasia was also a mosaic for TS (85% XO; 15% XX) (13).

The consensus based on the currently available evidence is that the intelligence level of TS patients is normal. There does not appear to be an increased incidence of moderate or severe mental retardation in these patients, nor do the individuals differ from their siblings in overall intelligence level (14). On the other hand, a characteristic neurocognitive and psychosocial profile has been described in TS females (3). Typically, specific deficits in visual-spatial/perceptual abilities, nonverbal memory function, motor function, executive function, and attentional abilities occur in TS children and adults of varying races and socioeconomic status (2,3). TS-associated psychosocial difficulties appear to occur in the areas of maturity and social skills. Our patient had difficulties in language skills and fine-motor coordination.

To our knowledge, this is the first report of a case of TS with concomitant THI and central diabetes insipidus in the literature. Further observations are needed in order to disclose the cause of THI and that of central diabetes insipidus in patients with TS.

## Figures and Tables

**Table 1:**
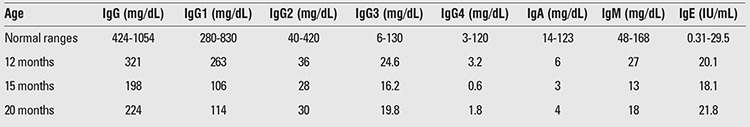
Serum immunoglobulin levels in the patient
